# Changes in dietary pattern in 15 year old adolescents following a 4 month dietary intervention with school breakfast – a pilot study

**DOI:** 10.1186/1475-2891-5-33

**Published:** 2006-12-07

**Authors:** Anne S Ask, Sigrunn Hernes, Ingebjørg Aarek, Gaute Johannessen, Margaretha Haugen

**Affiliations:** 1 Agder University College, Kristiansand, Norway; 2 Lista Secondary School, Lista, Norway; 3 Norwegian Institute of Public Health, P.O 4404, Nydalen, 0403 Oslo, Norway

## Abstract

**Background:**

Few studies on impact of meals served in school have been published. However, implications of school meals are an actual issue of both public and political concern in several countries. The objective of this study was to evaluate if breakfast served in a lower secondary school could improve dietary habits and school performance among the students.

**Methods:**

All students in 10^th ^grade in a lower secondary school, consisting of two school classes, were invited to participate in a controlled study. The students in one class were offered a free breakfast at the beginning of each school day for 4 months, while the students in the second class were controls. Both classes were educated in the importance of healthy eating, and a data program enabling them to evaluate dietary intake was introduced. The students answered two questionnaires, one on school performance and one short food frequency questionnaire, four weeks before study start and one week after. Body weight and height were measured by the school nurse at the beginning and end of the study. Because of few students in each group, non-parametrical statistic analyses were used.

**Results:**

All students in the intervention group had breakfast at school during the intervention. One week after the intervention the students in the class who received breakfast had returned to their normal breakfast pattern. In the control group the frequency of a lunch intake had increase, as compared to before study start (p < 0.01). An improved food pattern was seen among the male students in the intervention group, as measured by a healthy eating index after the intervention (p < 0.01). Body Mass Index increased statistically significant in both males and females in the control group (p < 0,01 for males and p < 0.05 for females), but not in the intervention group. Improvement in school performance following school breakfast was not found, but the males in the intervention group reported a significant increase in school contentment (p < 0.05).

**Conclusion:**

In a lower secondary school class served breakfast for 4 months, dietary intake changed to a more healthy profile and weight gain was reduced.

## Background

Evidence suggests that breakfast consumption and food habits have an impact on cognitive function and school performance among school children [1,2]. In two different studies Schoenthaler *et al*. showed that both antisocial behaviour and nonverbal intelligence improved in malnourished school children receiving a low-dose vitamin and mineral food supplement for 3–4 months [3,4]. So far cognitive improvement has been documented in undernourished populations, whereas, little or no improvement has been achieved in well nourished populations [5]. However, in a study among young adult prisoners antisocial behaviour and violence were reduced following vitamin, mineral and essential fatty acid supplementations for at least 2 weeks [6]. Furthermore, a study showed that intake of a low glycaemic index breakfast improved learning performance in young adults [7].

Adolescence is a time of transition and results often in a weakening of dietary habits in a period with search of independence [8]. In Norway, the schools do not provide breakfast or lunch, and hence these meals are based upon good parental follow-up. In a nationwide dietary survey among adolescents 15 – 20% stated that they did not eat breakfast and 55 – 60% did not eat lunch [9]. However, most lower secondary schools have a cafeteria alternative were the students can buy lunches. The food selection mostly consists of bread, sweet-buns, waffles, milk, soft drinks and fruit.

In a rural school situated in the southern part of Norway the teachers observed poor school attention and poor school performance, as well as antisocial behaviour among students in 10^th ^grade. The teachers suspected that poor dietary habits were a part of the complex. The objective of this study was to evaluate if dietary habits and school performance improved in a lower secondary school class as a result of introducing breakfast for 4 months.

## Methods

### Study design

Five weeks before study start the parents were invited to an evening meeting where oral and written information about the study was given. The importance of breakfast and a packed lunch for the students' cognitive performance was stressed. Practical information about nutritious packed lunches was also given. Four weeks before study start all students in 10^th ^grade were given information about the study and invited to participate. During one school hour all students were informed about the importance of a healthy diet for growth, learning performance and development. The students were asked to answer two questionnaires the same day. The same questionnaires were answered one week after the 4 months with intervention.

In the beginning of January 2005 one of the two classes, consisting of 26 students, were randomly assigned to the intervention group. The intervention consisted of served breakfast at the beginning of each school day. The students were also offered a food supplement consisting of vitamins, minerals and omega-3 fatty acids. The breakfast consisted of low fat milk, orange juice, whole grain bread, different spreads with fish, meat and cheese and a fruit. A conscientious objector working at the school was responsible for the preparation and serving of the breakfast. The other school class was not served breakfast, but got the same information about the importance of a healthy diet; in addition all parents were encouraged to provide a packed lunch for their children every day. One and three months into the intervention period all students were given one hour training in a data program, which enabled the student to evaluate their own diet[10].

Height (H) and weight (W) were measured with standard equipment by the school nurse before and after the study. Body Mass Index (BMI) was calculated by BMI = W(kg)/H^2^(m^2^). Overweight and obesity was categorized according to the definition established by Cole *et al*.[11]. A blood sample was drawn for haemoglobin concentration measurements before and after the study.

### Subjects

All students in 10^th ^grade in a secondary school, 54 adolescents at age 15, participated in this controlled intervention study with school breakfast for 4 months. The 10^th ^grade was divided into two classes, and the class allocated to a free school breakfast consisted of 15 males and 11 females. The control class had 14 males and 14 females. The secondary school is situated in a rural district in the southern part of Norway. Informed written consent was obtained from all parents and students. The study was approved by the Regional Ethical Committee and by the national Data Inspectorate.

### The questionnaires

The food frequency questionnaire (FFQ) was a non-validated questionnaire, which covered frequency intake of 27 food items commonly used in the Norwegian diet. Weekly intake of breakfast, lunch, dinner, evening meal and snack meals were also asked for in this questionnaire. Eleven food items from the food frequency questionnaire were chosen to calculate a healthy eating index. Frequent intake of two types of full bran breads, two types of low fat milk, boiled potatoes, vegetables and fruit were given a high score, whereas, frequent intake of white bread, whole fat milk, soft drinks, sweets and chocolates were given a low score, Table 1. Frequent intake of healthy food items were given the highest score 18, and frequent intake of unhealthy food items were given the lowest score 1. Missing data was replaced by the variable mean.

**Table 1:** Scores given to intake of eleven food items. The scores were added to calculate a healthy eating index.

**Food item**	**Intake: Frequency intake and scores**
Whole meal bread	Less than 4 times a week = 6 Between 4–6 times a week = 12 More than 7 times a week = 18
Whole meal crisp bread	Less than 4 times a week = 6 Between 4–6 times a week = 12 More than 7 times a week = 18
White bread	Less than 4 times a week = 6 Between 4–6 times a week = 4 7 and more times a week = 2
Two questions for two types of low fat milk	Never/seldom = 4 1–3 glasses a month = 5 Between 1–6 times a week = 10 Between 1–3 glasses a day = 15 4 and more glasses a day = 12
Whole fat milk	Never to 3 glasses a month = 8 Between 1–6 times a week = 4 1 and more glasses a day = 2
Sugar sweetened soft drinks	Never to 3 glasses per week = 4 4 times a week and more = 1
Boiled potatoes for dinner	Never to 4 times a week = 6 5 times a week and more = 12
Raw vegetables	Never to 4 times a week = 6 5 times a week and more = 12
Sweets	Never to 3 times a week = 6 3–4 times a week = 4 5 times a week and more = 1
Chocolates	Never to 3 times a week = 6 3–4 times a week = 4 5 times a week and more = 1

The questions in questionnaire two was chosen from a validated questionnaire and covered the students evaluation of the class environment, own performance and school satisfaction [12]. The teachers were asked to rate social behaviour, i.e. school attention and punctuality, in the two classes at the beginning and at the end of the study.

### Data analyses

Descriptive data is given as median (range) because of small groups (11–15 students in each group) and non-normality of continuous data in some groups. For calculations between group differences, Mann-Whitney test was employed and to test within group differences, Wilcoxon Signed Rank test was used. Statistical significance was set at p < 0.05. Data was analyzed employing SPSS statistical software (SPSS for Windows, version 13.01; SPSS Inc., Chicago, IL, USA).

## Results

### Anthropometric measurements

There was no statistical difference with regard to weight, height or BMI between the two classes at the start of the intervention, Table 2. Before study start two students were overweight and one was obese in the intervention group, whereas, two students were overweight in the control group. One student in the intervention group went from overweight to obesity; otherwise there was no change in the number of students with overweight or obesity. After the intervention period weight and BMI had increased significantly in both males and females in the control group (p < 0.01 for weight and p < 0.05 for BMI). There was also a significant increase in weight in the males in the intervention group (p < 0,05), but not in the females. BMI did not change significantly in the intervention group. No changes were measured in haemoglobin concentration as a result of the intervention, and none of the students had a haemoglobin concentration value below 11.0 g/100 mL.

**Table 2:** Weight, height, BMI and Haemoglobin given as median (range), in lower secondary school students participating in a study evaluating breakfast serving in school.

	Breakfast group		Control group	
	
	At start	After intervention	At start	After intervention
Height, m				
Males	177 (164–186)	179 (166–187)	175 (162–185)	178 (164–187)
Females	164 (154–179)	164 (154–179)	165 (158–174)	166 (159–175)
Weight, kg				
Males	73 (55–109)	73 (57–111)*	67 (50–90)	70 (54–92)**
Females	64 (49–81)	60 (48–76)	59 (45–77)	61 (48–80)**
BMI, kg/m^2^				
Males	22,6 (17,8–33,6)	21,8 (17,6–33,9)	21,7 (17,0–29,4)	22,4 (18,6–29,2)*
Females	21,8 (16,9–27,3)	22,1(17,5–28,1)	21,6 (16,7–28,4)	22,1 (16,9–28,7)*
Hemoglobin g/100 mL				
Males	15,3 (13,4–17,6)	14,9 (12,9–16,4)	14,4 (13,2–15,6)	14,7(13,2–15,8)
Females	13,6 (12,0–14,4)	14,1 (12,4–15,0)	13,3 (11,9–14,0)	13,1 (11,0–15,1)
Food score				
Males	69 (51–97)	85 (48–107)**	75 (57–93)	82 (56–97)
Females	73 (53–89)	81 (56–100)	79 (57–90)	79 (53–107)

Wilcoxon signed ranked test is used to calculate differences in the groups after 4 months intervention. *p < 0.05, ** p < 0.01

### Meal pattern

Before study start 14 students (54%) in the breakfast group and 12 (43%) in the control group had breakfast every day. During the intervention period almost all students in the breakfast group had breakfast every day at school. However, one week after the intervention period, the students in the intervention group went back to the breakfast habits from before the intervention. In the control group 3 (10%) of the students had increased breakfast frequency as reported one week after the intervention period. In the intervention group 52% of the students reported that they usually ate lunch every day before the intervention, and 58% reported lunch every day one week after. The responding figures in the control group were 81% and 86%. Intake of lunch in the control group was significantly higher compared to intake of lunch in the intervention group (p < 0.01). Furthermore, the increase in lunch frequency was statistically significant in the control group (p < 0.01) after the four months, while there was a non significant increase in the intervention group.

The healthy eating index increased significantly in the male student in the breakfast group (p < 0.01), whereas, there was a non significant increase in the control group. The intake of food supplements did not increase during the intervention period, which indicated that most of the students did not take advantage of the food supplement availability at breakfast.

### School environment

Students rating of the school environment did not improve as a result of the breakfast offering, but the males in the intervention group reported increased satisfaction with school (p < 0.05). School performance as measured by time spent doing home-work, did not increase as a result of the intervention.

The teachers did report an improvement in school attention and social behaviour in the breakfast group, but the improvement was not statistically significant, mostly because too few teachers were involved.

## Discussion

In this study we found that implementation of breakfast in a lower secondary school class was well received by the students and almost all students took advantage of this service every day. In the male group we found a significant improvement in a healthy eating index and in school satisfaction following the intervention, whereas, the improvement seen in the females did not reach statistical significance. We found no increase in BMI in the breakfast group during the study period, whereas, BMI increased significantly in the control group. Hence, mere information about healthy eating did not have a significant impact on dietary habits.

The finding that BMI did not increase statistically significant in the breakfast group during the study period, compared with the control group is noteworthy in this small pilot study. It is known that body weight has increased in Norwegian school children the last decade [13], and it is also shown that poor nutrient and energy intake in the morning contribute to an increased body weight [14-17]. Introducing a breakfast meal in school might be one way to fight obesity among adolescence and young adults.

Changes in school performance as a result of breakfast offering were not found in this study. This is in accordance with results from other studies, where dietary manipulations has been investigated in well nourished children [2,18], whereas, improvement has been reported in low-income communities [2,19,20]. However, the shortcomings of this study, i.e. short time period and the small sample size, gives this pilot study too little statistical power to detect small to mediocre improvement. The teachers reported improvement in school attention among the students in the intervention group, but there were too few teachers involved in the two classes to make it possible to calculate statistical differences. Furthermore, the students were asked about their own school performance, and no objective measurement was included, because the investigators had not asked for permission on beforehand to examine the schools' evaluation of the students.

For this project a special lower secondary school was investigated, because the teachers asked for assistance with poor school attendance and antisocial behaviour among the students. This might have had an impact on the results, because the antisocial pattern was already well established and a more comprehensive strategy would have been required to break the pattern. The teachers had already tried out different disciplinary strategies without any luck, and hence the breakfast serving was a last attempt to improve the class situation. The teachers, however, did not attend the school breakfast, and gave little attention to the project. This might have contributed negatively to the result, but on the other hand if a breakfast meal would have major impact on school performance, there was room for improvement among these 10^th ^graders.

Several important factors seemed to have failed during the completion of the study; firstly the conscientious objector was not well enough trained for the project and the implementation of food supplement failed. As a consequence hardly any students complied with this part. Secondly, serving of fruit was not done as instructed. Whole fruit was served instead of fruit cut into slices. Intake of fruit has been documented to increase if the fruit is served in slices [21]. The students reported satisfaction with the breakfast meal and reported that they sadly missed the morning meal when no longer available. However, the teachers were not all satisfied with the practical solution of the breakfast serving, because the breakfast had to be served and eaten in the classroom, which resulted in a delay in the first school lesson. The breakfast setting would have been better if breakfast could have been served in the cafeteria or at least in a separate room.

This was a small pilot study performed in an attempt to improve dietary habits, school attention and social behaviour among students in a lower secondary school. In this study we saw improved food intake, reduction in weight gain and increased satisfaction with the school day, but the study suffers from too little statistical power. Increasing the statistical power by increasing the number of students, improving the methods to measure social behaviour and being able to objective measurements of school performance should be applied to future studies.

## Conclusion

This study shows that breakfast offered to students in a secondary school class for 4 months improved dietary habits and reduced the weight gain. This could imply that a school meal could have major impact on health later in life. However, because the study had too little statistical power, evidence for improve school performance and social behaviour in well nourished children could not be established.

## Competing interests

The author(s) declare that they have no competing interests.

## Authors' contributions

ASA, SH, IA, GJ and MH have all given substantial contribution to the study concept and design. ASA, SH and IA carried out the practical part and supervised the conscientious objector. All authors have contributed to drafting and critically revised the manuscript. MH has written the manuscript and performed the statistical calculations.
